# SARS-CoV-2 N Gene G29195T Point Mutation May Affect Diagnostic Reverse Transcription-PCR Detection

**DOI:** 10.1128/spectrum.02223-21

**Published:** 2022-01-12

**Authors:** Karrie K. K. Ko, Nurdyana Binte Abdul Rahman, Shireen Yan Ling Tan, Kenneth X. L. Chan, Sui Sin Goh, James Heng Chiak Sim, Kun Lee Lim, Wan Loo Tan, Kian Sing Chan, Lynette L. E. Oon, Niranjan Nagarajan, Chayaporn Suphavilai

**Affiliations:** a Department of Microbiology, Singapore General Hospital, Singapore; b Department of Molecular Pathology, Singapore General Hospital, Singapore; c Genome Institute of Singapore, Genome Institute of Singapore, A*STAR, Singapore; d Yong Loo Lin School of Medicine, National University of Singapore, Singapore; e Duke-NUS Medical School, National University of Singapore, Singapore; University of Cincinnati

**Keywords:** diagnostic escape, G29195T, N gene, SARS-CoV-2

## Abstract

Rapid onsite whole-genome sequencing of two suspected severe acute respiratory syndrome coronavirus 2 (SARS-CoV-2) N gene diagnostic escape samples revealed a previously unreported N gene point mutation at genome position 29195. Because the G29195T mutation occurs within a region probed by a commonly referenced U.S. CDC N gene reverse transcription (RT)-PCR assay, we hypothesize that the G29195T mutation rendered the N gene target of a proprietary commercial assay undetectable. The putative diagnostic escape G29195T mutation demonstrates the need for nearly real-time surveillance, as emergence of a novel SARS-CoV-2 variant with the potential to escape diagnostic tests continues to be a threat.

**IMPORTANCE** Accurate diagnostic detection of SARS-CoV-2 currently depends on the large-scale deployment of RT-PCR assays. SARS-CoV-2 RT-PCR assays target predetermined regions in the viral genomes by complementary binding of primers and probes to nucleic acid sequences in the clinical samples. Potential diagnostic escapes, such as those of clinical samples harboring the G29195T mutation, may result in false-negative SARS-CoV-2 RT-PCR results. The rapid detection and sharing of potential diagnostic escapes are essential for diagnostic laboratories and manufacturers around the world, to optimize their assays as SARS-CoV-2 continues to evolve.

## OBSERVATION

Reliable diagnostic detection of severe acute respiratory syndrome coronavirus 2 (SARS-CoV-2) is critical for clinical management and containment of coronavirus disease 2019 (COVID-19). Reverse transcription (RT)-PCR assays of SARS-CoV-2 RNA have been widely deployed since the beginning of the COVID-19 pandemic (1). The performance of these assays is dependent on the complementary binding of predetermined primers and probes to targeted sequences in the viral genome. For SARS-CoV-2, E, N, S, RNA-dependent RNA polymerase (RdRp), and open reading frame 1ab (ORF1ab) regions are common targets in both laboratory-developed tests (LDTs) and commercial assays (2, 3). The expected molecular evolution in SARS-CoV-2 genomes (4) means that variants will continue to emerge. This can result in mutations leading to RT-PCR diagnostic escapes and false-negative RT-PCR results. As a result, our College of American Pathologists (CAP)-accredited diagnostic laboratory has validated four LDTs and commercial assays for routine SARS-CoV-2 testing, to ensure that a range of gene targets are available for diagnostic utility and confirmation even as SARS-CoV-2 viral genomes continue to evolve. We have since performed more than 479,000 SARS-CoV-2 tests in our laboratory.

Multiple diagnostic escapes have been reported. Three N gene point mutations, i.e., C29200T (5), G29140U (6), and C29200A (7), and one N gene 6-nucleotide deletion at genome position 28889 (8) have been reported to affect N gene detection in RT-PCR assays. Here, we report a novel N gene point mutation, G29195T, which affected the detection of the SARS-CoV-2 N gene by the Cepheid Xpert Xpress SARS-CoV-2 assay.

The Xpert assay is an FDA-approved assay for COVID-19 under emergency use authorization (EUA). The proprietary multiplex RT-PCR assay targets both the E gene and the N gene. Since its implementation in our laboratory in May 2020, 111,883 samples have been tested with the Xpert assay, of which 4,762 (0.99%) were positive. In October 2021, we received two nasopharyngeal swab samples from an ambulatory clinic. Both samples were strongly positive for the E gene, while the N gene was not detected by the Xpert assay (Table 1). Two other validated tests in our laboratory confirmed these samples to be true-positive samples, with the E gene, ORF1ab, and the S gene being consistently detected (Table 1). This study involved the use of remnant clinical specimens from two anonymous patients and did not require institutional review board (IRB) review, according to SingHealth centralized IRB policy.

**TABLE 1:** Summary of RT-PCR results for two clinical samples with suspected N gene diagnostic escape and N gene mutations detected

Assay and target region or sample name	Cycle threshold value		N gene mutations^*a*^
	SNDE1	SNDE2	
Xpert Xpress SARS-CoV-2 assay (Cepheid, USA)			
E gene	12.9	13.3	
N gene	Not detected	Not detected	
cobas SARS-CoV-2 test on cobas 6800 system (Roche Diagnostics, Switzerland)			
E gene	15.66	Not done^*b*^	
ORF1ab	15.46	Not done^*b*^	
RealStar SARS-CoV-2 RT-PCR kit v1.0 (altona Diagnostics GmbH, Germany)			
E gene	12.01	12.37	
S gene	11.62	11.83	
SNDE1			PANGO lineage (11): AY.23.1 (sublineage B.1.617.2); SNPs: A28461G, G28881T, G28916T, G29195T, G29402T; deletions/insertions: none in N gene (genome positions 28274–29533); amino acid substitutions: D63G, R203M, G215C, A308S, D377Y
SNDE2			PANGO lineage (11): AY.23.1 (sublineage B.1.617.2); SNPs: A28461G, G28881T, G28916T, G29195T, G29402T; deletions/insertions: none in N gene (genome positions 28274–29533); amino acid substitutions: D63G, R203M, G215C, A308S, D377Y

a Compared to the reference sequence (GenBank accession number NC_045512). SNP, single-nucleotide polymorphism.

b There was insufficient remnant for sample SNDE2 for the Roche cobas SARS-CoV-2 test to be performed.

Whole-genome sequencing of samples SNDE1 (GenBank accession OL413478) and SNDE2 (GenBank accession OL413479) was performed as part of a SARS-CoV-2 surveillance sequencing program in our laboratory. Total nucleic acid extraction of the chemically inactivated remnant samples was performed on a Maxwell RSC 48 instrument, using the Maxwell RSC viral total nucleic acid purification kit (Promega, USA). The resultant total nucleic acid extract was used for downstream RT-PCR and sequencing on a MinION system (Oxford Nanopore Technologies, Oxford, UK) in accordance with the ARTIC protocol v3 (9). The RAMPART protocol (10) was used to monitor the depth of coverage for each sample and to construct a draft genome.

**FIG 1:**
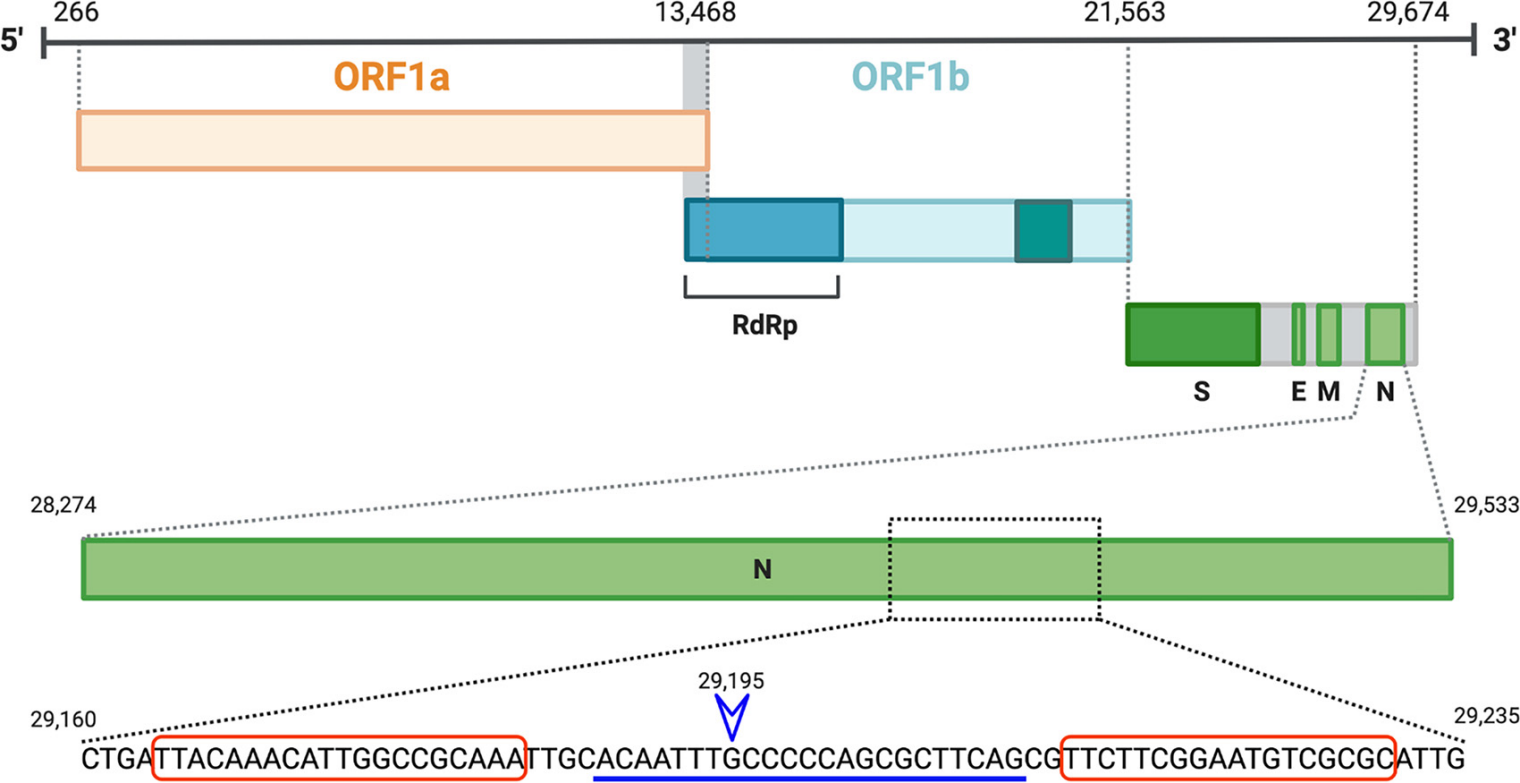
Diagrammatic representation of the SARS-CoV-2 N gene. Red boxes denote regions targeted by CDC N2 assay primers. The blue line denotes the region targeted by the CDC N2 assay probe (2). A point substitution from G to T at genome position 29195 (blue arrowhead) was detected in samples SNDE1 and SNDE2.

A point mutation, G29195T, was detected in the consensus sequences of SNDE1 and SNDE2 but not in other surveillance samples sequenced in our laboratory between June 2020 and October 2021 (*n* = 700). Table 1 summarizes the N gene mutations detected in SNDE1 and SNDE2. No previously reported mutation contributing to N gene diagnostic escape was detected. G29195T was suspected to affect N gene detection because this mutation lies within a region targeted by one of the U.S. CDC assays (N2) (2) (Fig. 1). This is in keeping with previously reported findings (5, 7) that a single point substitution within the CDC N2 probe binding region was sufficient to impair N2 gene detection in some assays.

This G29195T mutation is a novel mutation in Singapore and was first reported in the GISAID database (https://www.gisaid.org) in October 2021. Within 5 weeks, an additional four SARS-CoV-2 genome sequences with this mutation were reported from Singapore, excluding the two samples described in this report. Worldwide, of >4.9 million SARS-CoV-2 genomes uploaded to the GISAID database, 2,617 genomes harbor this mutation. Although this is a small percentage, this is a reminder that the emergence of a novel SARS-CoV-2 variant with the potential to escape diagnostic tests continues to be a threat. Therefore, SARS-CoV-2 diagnostic assays should ideally be based on two or more gene targets. A robust molecular surveillance system is necessary to monitor for emergent diagnostic escapes.

### Data availability.

Sequence data were submitted to GenBank with the accession numbers OL413478 (SNDE1) and OL413479 (SNDE2).
